# The influence of coach leadership behavior on athletes’ prosocial behavior in sports: The mediating effect of goal orientation and moral disengagement in sports

**DOI:** 10.1371/journal.pone.0346829

**Published:** 2026-05-29

**Authors:** Xiangui Bu, Zeqi Huang, Shuaiwei Zheng, Yi Zhang, Shasha Ma, Yuliang Kang

**Affiliations:** 1 Department of Sports Training, Shandong Sport University, Rizhao, Shandong, China; 2 Department of Graduate Education, Shandong Sport University, Jinan, Shandong, China; 3 Department of Physical Education, Shandong Sport University, Rizhao, Shandong, China; Julius-Maximilians-Universität Würzburg: Julius-Maximilians-Universitat Wurzburg, GERMANY

## Abstract

To investigate the impact of coach leadership behavior on athletes’ prosocial behavior in sports, as well as the mediating role of goal orientation and moral disengagement in it, a questionnaire survey was conducted on 1239 athletes. The results showed that democratic leadership behavior has significant positive and negative effects on athletes’ prosocial behavior and antisocial behavior in sports, respectively; Authoritarian leadership behavior has significant negative and positive impact on athletes’ prosocial behavior and anti social behavior in sports, respectively; Both democratic leadership behavior and authoritarian leadership behavior can have a significant indirect impact on prosocial behavior and antisocial behavior in sports through the mediating effect of goal orientation and moral disengagement. Specific mediating paths include independent mediating paths of self-orientation, task orientation, and moral disengagement in sports, and chain mediating paths of self-orientation and moral disengagement in sports. In the future, coaches can enhance democratic leadership, reduce authoritarian leadership behavior, adjust athletes’ goal orientations, and lower moral disengagement levels, thereby suppressing antisocial behavior and promoting prosocial behavior in sports.

## 1 Preface

As the main body of competitive sports, athletes’ moral behavior affects the national image and international reputation. However, due to the fierce competition in competitive sports and athletes’ pursuit of utilitarian results, moral misconduct among athletes often occurs, which has a negative impact on the healthy development and dissemination of sports competitions. In sports psychology research, sports moral behavior is usually divided into sports prosocial behavior and sports antisocial behavior based on the impact of individual athlete behavior on others [1,2]. Sports prosocial behavior refers to the voluntary behavior exhibited by individual athletes to help or benefit others; Sports antisocial behavior refers to voluntary behavior by athletes that harms or puts others at a disadvantage [3]. Studies have shown that the prosocial behaviors of athletes during their sports activities contribute to better performance, while the antisocial behaviors of athletes are detrimental to their positive performance. (Al Yaaribi et al., 2016; Al Yaaribi& [4,5]. Therefore, it is particularly important to promote athletes’ prosocial behavior in sports and prevent or reduce antisocial behavior in sports.

Trigueros et al.’s [6] research has found that the self-directed coaching style significantly predicts antisocial behavior in sports; The controlled coaching style significantly predicts antisocial behavior in sports. From this, it can be seen that coaches are an undeniable factor influencing athletes’ prosocial behavior in sports. Zhu et al.’s [7] research has found that moral disengagement in sports can positively predict antisocial behavior in sports, but is not significantly correlated with prosocial behavior in sports. Mallia et al. [8] found that the significant positive prediction of antisocial behavior and a negative prediction of prosocial behavior in sports ethics. Yukhymenko-Lescroart [9] and Lucidi et al. [10], the goal orientation includes two dimensions: self-orientation and task orientation. Among them, self-orientation can significantly positively predict the moral disengagement of sports, while task orientation cannot significantly predict the moral disengagement of sports, Kavussanu [11] in the process of exploring the relationship between goal orientation and prosocial behavior of football players, it was found that task orientation is a positive predictor of prosocial behavior and a negative predictor of antisocial behavior; Self-orientation is a positive predictor of antisocial behavior and a negative predictor of prosocial behavior. From this, it can be seen that both moral disengagement and goal orientation in sports are related to pro-antisocial behavior in sports, and there is also an important correlation between moral disengagement and goal orientation in sports. However, the mechanisms by which goal orientation and sports ethics disengagement affect the leadership behaviors of coaches and the antisocial behaviors of athletes remain unclear. In view of this, this study examines the impact and pathway of coach leadership behavior on athlete prosocial behavior, and tests the mediating role of goal orientation and sports ethics disengagement between the two, in order to further clarify the value of coach leadership behavior, athlete goal orientation, and sports ethics disengagement, in order to provide theoretical guidance for promoting athlete prosocial behavior in sports and preventing or reducing sports antisocial behavior. It should be noted that since this article focuses on the perspective of athletes, the coaching leadership behavior referred to in this article refers to the “coaching leadership behavior perceived by athletes.”

## 2 Literature review and research hypotheses

### 2.1 The direct effect of coach leadership behavior on pro-antisocial behavior in sports

Coach leadership behavior refers to the various behaviors exhibited by coaches in the process of influencing athletes through their own words and actions [12]. Some scholars have conducted research on coaching leadership styles by using the terms “democratic” and “authoritarian.” [13]. Democratic leadership behavior refers to coaches providing athletes with more opportunities to participate in the collective decision-making process; Authoritarian leadership behavior refers to a top-down management style where everything is decided by the coach and the athletes follow those decisions [14]. Si et al. [15] emphasize the roles of coaches, part-time mentors, and parents. Research has shown that democratic parenting styles are positively correlated with social responsibility, while authoritarian parenting styles are negatively correlated with social responsibility [16,17]. Lee et al.’s [18] research has found that coach democratic behavior is significantly positively correlated with athletes’ social responsibility, while authoritarian behavior is significantly negatively correlated with athletes’ social responsibility. Based on the above literature, it can be inferred that coach democratic leadership behavior can enhance athletes’ level of responsibility, effectively promote ethical behavior norms among athletes, and reduce the occurrence of moral misconduct; The authoritarian leadership behavior of coaches will weaken the level of responsibility of athletes, which in turn is not conducive to their compliance with ethical norms and more unethical behavior. Based on this, this study proposes a hypothesis: H1a Coach’s democratic leadership behavior significantly positively predicts prosocial behavior in sports; H1b Coach’s democratic leadership behavior significantly negatively predicts antisocial behavior in sports; H1c Coach’s authoritarian leadership behavior significantly negatively predicts prosocial behavior in sports; H1d Coach’s authoritarian leadership behavior significantly positively predicts antisocial behavior in sports.

### 2.2 Mediating effects and hypotheses of target orientation

The definition of goal orientation is the purpose of action, but these orientations are also considered to be more persistent engagement tendencies [19]. It is divided into two dimensions: task orientation and self-orientation. Task orientation focuses on the mastery and improvement of skills, indicating that individuals must work hard to learn and cooperate with others in order to succeed; Individual orientation focuses on objective performance outcomes and interpersonal comparisons, indicating that individuals need to establish goals that are superior to others, and believe that in order to succeed, they need to strive to defeat others and possess superior abilities [19,20]. Koh and Wang [21] researched that coach behavior can influence athletes’ goal orientation and participation motivation from multiple perspectives. Syrmpas et al.’s [22] research has found that task-oriented athletes believe that their coaches exhibit less verbal aggression, authoritarianism, and more democratic behavior; Self oriented athletes believe that their coaches exhibit more verbal aggression, authoritarianism, and less democratic behavior. Based on the achievement goal theory, Kavussanu [2] in the process of exploring the relationship between goal orientation and prosocial behavior of football players, it was found that task orientation is a positive predictor of prosocial behavior and a negative predictor of antisocial behavior; self-orientation is a positive predictor of antisocial behavior and a negative predictor of prosocial behavior. Meanwhile, scholars have confirmed that both dimensions of goal orientation (self-orientation and goal orientation) can be studied as mediating variables [23,24]. Based on the above literature, it can be inferred that coach democratic behavior can promote athletes’ prosocial behavior and prevent or reduce antisocial behavior by enhancing their task orientation and weakening their self-orientation; Coach authoritarian behavior can reduce athletes’ task orientation, enhance their self-orientation, thereby reducing their prosocial behavior in sports and increasing their antisocial behavior in sports.

Based on this, this study proposes a hypothesis: H2a There is a mediating effect of self-orientation between coach’s democratic behavior and athlete’s prosocial behavior in sports; H2b Task orientation plays a mediating role between coach democratic behavior and athlete prosocial behavior in sports; H2c There is a mediating effect of self-orientation between coach democratic behavior and athlete antisocial behavior in sports; H2d Task orientation mediates the relationship between coach democratic behavior and athlete antisocial behavior in sports; H2e There is a mediating effect of self-orientation between coach authoritarian behavior and athlete prosocial behavior in sports; H2f Task orientation plays a mediating role between coach authoritarian behavior and athlete prosocial behavior in sports; H2g There is a mediating effect of self-orientation between coach authoritarian behavior and athlete antisocial behavior in sports; H2h Task orientation plays a mediating role between coach authoritarian behavior and athlete antisocial behavior in sports.

### 2.3 The mediating effect and hypothesis of moral disengagement in sports

Moral disengagement in sports refers to specific cognitive tendencies developed by individual athletes, including cognitive restructuring of their behavior to minimize harm, minimizing responsibility for the consequences of their actions, and reducing identification with the injured [25]. Democratic coaches allow athletes to participate in decision-making and actively encourage them to offer training suggestions or competition strategies. This makes the athletes feel valued and respected, and also sets an ethical example for them, promoting the formation of their sense of responsibility and the shaping of their social responsibility. Therefore, athletes are unlikely to let their own sports ethics take the lead in order to win during training and competition. Authoritarian coaches do not allow athletes to participate in decision-making, often guiding training and competition in a commanding tone, and requiring athletes to unconditionally accept and obey the coach’s opinions. This leads to athletes ignoring ethical behavior norms in order to gain an advantage in training and competition or meet the coach’s requirements, while reducing their sense of responsibility and empathy.

In the research on the relationship of antisocial behavior, it was found that scholars unanimously agreed that the evasion of sports ethics is a positive predictor of antisocial behavior in sports [26,27], but there are differences in the exploration of the relationship between sports moral disengagement and sports prosocial behavior. One viewpoint suggests that moral disengagement in sports is a negative predictor of prosocial behavior in sports [28]. Another viewpoint suggests that moral disengagement in sports is not a significant predictor of prosocial behavior in sports [26]. According to social cognitive theory and corresponding research results [8,28], this study suggests that an individual’s cognitive evaluation results can have an impact on their psychology and behavior.

Based on the above literature, this study proposes a hypothesis: H3a There is a mediating effect of moral disengagement in sports between coaches’ democratic behavior and athletes’ prosocial behavior in sports; H3b There is a mediating role of moral disengagement in the relationship between coach democratic behavior and athlete antisocial behavior in sports; H3c There is a mediating effect of moral disengagement in sports between coach authoritarian behavior and athlete prosocial behavior in sports; H3d There is a mediating effect of moral disengagement in sports between coach authoritarian behavior and athlete antisocial behavior in sports.

### 2.4 Chain mediation effect and hypothesis of goal orientation and moral disengagement in sports

Lucidi et al. [10] it is believed that task-oriented athletes are more inclined to compete fairly and justly based on their athletic skills and methods, and tend to avoid moral disengagement in sports; Self oriented athletes, on the other hand, are willing to do whatever it takes to win and interpret actions that harm their opponents as protecting their teammates or shifting responsibility to the coach. Yukhymenko- Lescroart’s [9] research has found that only the self-orientation dimension in goal orientation can effectively predict athletes’ moral disengagement in sports. Based on the above analysis, there is a close relationship between different coach leadership behaviors and various dimensions of athlete goal orientation. Athletes’ self-orientation affects their moral disengagement in sports, and moral disengagement in sports has a predictive effect on pro-antisocial behavior in sports.

**Fig 1:**
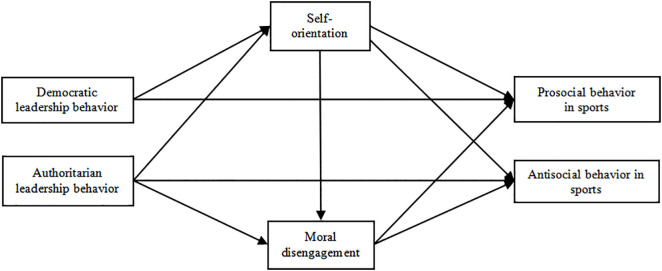
Assuming model 1.

**Fig 2:**
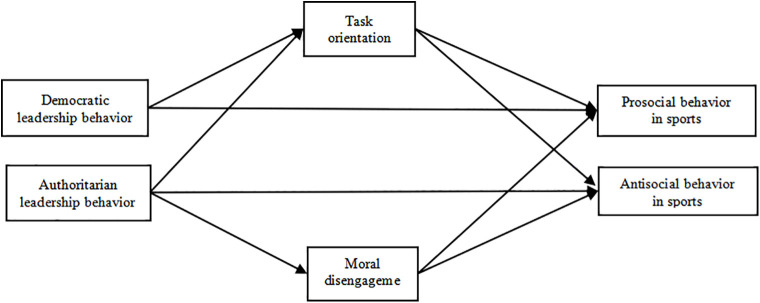
Assuming model 2.

Based on this, this study proposes a hypothesis: H4a There is a chain mediation effect between coach democratic behavior and athlete prosocial behavior in terms of self-orientation and moral disengagement in sports; H4b There is no chain mediation effect between task orientation and ethical disengagement in coaches’ democratic behavior and athletes’ prosocial behavior in sports; H4c There is a chain mediation effect between coach democratic behavior and athlete antisocial behavior in terms of self-orientation and moral disengagement in sports; H4d There is no chain mediation effect between coach democratic behavior and athlete antisocial behavior in terms of task orientation and ethical disengagement in sports; H4e There is a chain mediation effect between coach authoritarian behavior and athlete prosocial behavior in terms of self-orientation and moral disengagement in sports; H4f There is no chain mediation effect between coach authoritarian behavior and athlete prosocial behavior in terms of task orientation and moral disengagement in sports; H4g There is a chain mediating effect between coach authoritarian behavior and athlete antisocial behavior in terms of self-orientation and moral disengagement in sports; H4h There is no chain mediation effect between coach authoritarian behavior and athlete antisocial behavior in terms of task orientation and moral disengagement in sports. Therefore, the hypothesis model constructed in this study is shown in Figs 1 and 2:

## 3 Research objects and methods

### 3.1 Research object

The research object of this article is the influence of coach leadership behavior on athletes’ prosocial behavior in sports, as well as the mediating role of goal orientation and moral disengagement in sports. Using the G*Power3.1 Software to performs pre-sample size estimation and set α = 0.05, power(1-β)=0.8. When the effect size is set to medium (0.15), the results show that a sample size of at least 143 people is required. Therefore, in order to ensure that the sample size has sufficient statistical power, the recruitment process took place from 12/5/2024–20/10/2024. Through on-site investigations and during the breaks of the athletes’ competitions, a cluster sampling method was employed to select over 30 sports teams from the national training team of China and 10 provinces including Shandong, Henan, Anhui, Jiangsu, Zhejiang, Jilin, Shaanxi, Sichuan, Beijing, and Tianjin. The athletes (with a sports level of national second-class athlete or above) from these teams were selected as the subjects for the questionnaire survey. This research was conducted after obtaining approval from the Academic Ethics Committee of Shandong Sports University (Ethical Approval Number: 2023036). All participants signed an informed consent form, and the data were collected anonymously. All data were only used for experimental analysis. A total of 1,382 questionnaires were distributed, and 1,239 valid questionnaires were retrieved, with an effective recovery rate of 89.7%. Among them, there were 819 males (66.1%) and 420 females (33.9%); 693 were second-class athletes (55.9%), 460 were first-class athletes (37.1%), and 86 were national champion athletes (6.9%). The average age of the athletes was 20.10 years (SD = 2.54), and the average training duration was 5.93 years (SD = 2.65). The sports events included: boxing, taekwondo, wrestling, discus throwing, judo, gymnastics, volleyball, basketball, weightlifting, tennis, rugby, and canoeing, etc.

### 3.2 Measuring tools

#### 3.2.1 Coach leadership behavior scale.

The Coaching Leadership Behavior Scale are originally developed by Chelladurai et al. and subsequently revised by Yang Yong [29]. This scale consists of 10 questions, such as “involving athletes in the decision-making process” and “guiding training and competition with a commanding tone,” including 2 dimensions: democratic and authoritarian, with 5 questions in each dimension. Adopting a 5-point rating system, from 1 being “never” to 5 being “always,” the higher the score, the more obvious the leadership style. In this study, the dimensions are divided into Cronbach’s α The coefficients are 0.90 for democratic leadership and 0.85 for authoritarian leadership. The results of confirmatory factor analysis are:$\frac{\chi^{\text{2}}}{df}$=2.76, RMSER = 0.04, SRMR = 0.02, IFI = 0.99, CFI = 0.99, NFI = 0.99, TLI = 0.99, This indicates that the revised scale has good structural validity.

#### 3.2.2 Task orientation and self-orientation questionnaire in sports.

The “Task Orientation and self-orientation Questionnaire in Sports” [30] was developed using the principle of equal tension. This study made slight changes to the wording of the questionnaire stem (changing “classmate” to “teammate”). Secondly, exploratory factor analysis reveals that: KMO The value is 0.90, P = 0.000 < 0.001, The assignment of the rotated component matrix questions is the same as the original questionnaire, and the factor loads are all greater than 0.7. The questionnaire consists of 13 questions, such as “When I am at the best” and “When I train very hard,” including 2 dimensions: self-orientation and task orientation. Adopting a 5-point rating system, from 0 indicating ‘not at all’ to 4 indicating ‘completely,’ the higher the score, the stronger the goal orientation. In this study, the dimensions are divided into Cronbach’sα The coefficients are 0.81 (self-orientation) and 0.89 (task orientation). The result of confirmatory factor analysis is:$\frac{\chi^{\text{2}}}{\text{df}}$=2.97, RMSER = 0.04, SRMR = 0.04, IFI = 0.98, CFI = 0.98, NFI = 0.97, TLI = 0.98. This indicates that the revised questionnaire has good structural validity.

#### 3.2.3 Exercise moral deduction scale.

The study employed the Sport Moral Disengagement Scale developed by Boardley and Kavussanu [31]. The scale consists of 8 questions, such as’ It is not wrong for athletes to lie to referees in order to help their team ‘, which belongs to a single-dimensional scale. Adopting a 7-level rating system, from 1 being “strongly disagree” to 7 being “strongly agree,” the higher the score, the higher the degree of moral disengagement in sports. In this study, the Sports Ethics Deduction Scale Cronbach’s α coefficient is 0.77. The result of confirmatory factor analysis is:$\frac{\chi^{\text{2}}}{df}$=2.49, RMSER = 0.04, SRMR = 0.02, IFI = 0.99, CFI = 0.99, NFI = 0.99, TLI = 0.99, This indicates that the scale has good structural validity.

#### 3.2.4 Sports prosocial behavior scale.

The Kinssanu-developed and Zhu [32] revised “Physical Activity and Antisocial Behavior Scale” was adopted. This scale consists of 23 items, such as “encouraging teammates,” “providing help to injured opposing team members,” “insulting teammates,” and “intentionally causing injury to opposing team members,” including four dimensions: sports prosocial behavior (teammates) and sports prosocial behavior (opponents), sports antisocial behavior (teammates), and sports antisocial behavior (opponents). Using a 5-point rating system, from 1 for “never” to 5 for “very often,” the higher the score, the more frequently the athlete engages in corresponding behavior. In this study, the total scale Cronbach’s α coefficient is 0.84, which divides the dimensions of sports prosocial behavior (teammates), sports prosocial behavior (opponents), sports antisocial behavior (teammates), and sports antisocial behavior (opponents) Cronbach’s α coefficients are 0.83, 0.75, 0.85, 0.93. The result of confirmatory factor analysis is: = 3.72, RMSER = 0.05, SRMR = 0.04, IFI = 0.97, CFI = 0.97, NFI = 0.95, TLI = 0.96. This indicates that the revised scale has good structural validity.

### 3.3 Statistical methods

The questionnaire is distributed to athletes through the Wenjuanxing platform. Before answering the questions, the experimenter explains to the participants the confidentiality, anonymity, and filling requirements of the questionnaire to ensure the authenticity and validity of the data. Using SPSS26.0 and AMOS23.0 to organize and analyze data using statistical methods, including reliability and validity testing, confirmatory factor analysis, common method bias testing, descriptive analysis, analysis of variance, correlation analysis, regression analysis, and more Bootstrap analysis. This study is mainly based on Wen et al. [33], the latest mediation effect testing process proposed is used to test the research hypothesis.

## 4 Research results and analysis

### 4.1 Common method deviation test

Since the self-report questionnaire was adopted, all the data were derived from the self-reports of the athletes. To prevent the occurrence of common method bias, anonymous measures and other methods were used to collect the data. The Harman single-factor method recommended by Zhou and Long [34] was employed for the test. The specific approach was as follows: all the measurement items of the research variables were loaded onto only one common factor, a single-factor model was established, and then the fitting indices of the single-factor model and the 9-factor model that conformed to the theoretical dimensions were compared. The results showed that the fitting index of the 9-factor model (χ2df = 3.38, RMSER = 0.04, SRMR = 0.05, IFI = 0.91, CFI = 0.91, NFI = 0.88, TLI = 0.91) was significantly better than that of the single-factor model (χ2df = 14.70, RMSER = 0.11, SRMR = 0.13, IFI = 0.47, CFI = 0.47, NFI = 0.46, TLI = 0.45), indicating that there was no serious common method bias problem in the data of this study.

### 4.2 Analysis of the current situation of prosocial and antisocial behavior among athletes

The mean scores of prosocial behavior and antisocial behavior were 3.93 and 1.56 respectively (Table 1), indicating that, overall, the level of sports prosocial behavior among athletes was higher, while the level of sports antisocial behavior was lower. This study also examined whether there were differences in sports prosocial and antisocial behaviors in terms of gender and sports level. For sports prosocial behavior, the results showed that the gender main effect was not significant (F = 0.06, P = 0.81), the sports level main effect was not significant (F = 0.38, P = 0.69), and the interaction between gender and sports level was not significant (F = 1.37, P = 0.26). For sports antisocial behavior, the results showed that the gender main effect was significant (F = 6.63, P < 0.05), the sports level main effect was not significant (F = 2.19, P = 0.11), and the interaction between gender and sports level was not significant (F = 0.22, P = 0.80). In terms of gender, the total mean score of antisocial behavior for male athletes (X = 1.59) was significantly higher than that for female athletes (X = 1.49).

**Table 1:** List of Descriptive Statistics and Correlation Analysis for Each Variable (n = 1239).

	X	SD	1	2	3	4	5	6	7
1. Democratic leadership behavior	3.94	0.95	1.00						
2. Authoritarian leadership behavior	1.87	0.84	−0.38^***^	1.00					
3. Self-orientation	1.77	0.80	−0.21^***^	0.27^***^	1.00				
4. Task orientation	3.10	0.75	0.53^***^	−0.22^***^	−0.15^***^	1.00			
5. Moral disengagement in sports	3.17	1.10	−0.19^***^	0.38^***^	0.34^***^	−0.13^***^	1.00		
6. Prosocial behavior in sports	3.93	0.85	0.52^***^	−0.29^***^	−0.24^***^	0.58^***^	−0.21^***^	1.00	
7. Antisocial behavior in sports	1.56	0.67	−0.31^***^	0.51^***^	0.28^***^	−0.26^***^	0.44^***^	−0.25^***^	1.00

Note: * indicates P < 0.05, **Expressing P < 0.01, ***Expressing P < 0.001; the same below.

### 4.3 Correlation analysis of variables

The correlation analysis of various variables (Table 1) shows that there is a significant correlation between democratic leadership behavior, authoritarian leadership behavior, task orientation, self-orientation, moral disengagement in sports, antisocial behavior in sports, and prosocial behavior in sports; And the correlation coefficients between variables are all below 0.7, indicating that there is no collinearity problem between variables, and providing strong support for the mediation effect test in the following text.

### 4.4 Regression analysis of coach leadership behavior on pro antisocial behavior in sports

Using hierarchical regression, a regression analysis was conducted on the relationship between coach leadership behavior and sports prosocial behavior (Tables 2 and 3). The results showed that after controlling for demographic variables such as gender, age, years of exercise, and exercise level, democratic leadership behavior significantly positively predicted prosocial behavior (β = 0.52, P < 0.001) and significantly negatively predicted antisocial behavior (β = −0.32, P < 0.001), explaining 28% and 11% of the variance, respectively. In addition, authoritarian leadership behavior significantly negatively predicted prosocial behavior in sports (β = −0.30, P < 0.001) and significantly positively predicted antisocial behavior in sports (β = 0.51, P < 0.001), explaining 9% and 26% of the variance, respectively. Therefore, the research hypothesis H1a、H1b、H1c、H1d all of them are valid, indicating that coach democratic leadership behavior can effectively promote athletes to engage in prosocial sports behavior and less engage in antisocial sports behavior; The authoritarian leadership behavior of coaches can easily lead to the formation of antisocial behavior in athletes, and they are less likely to engage in prosocial behavior in sports.

**Table 2:** Regression Analysis of Democratic Leadership Behaviour on Prosocial and Antisocial Behaviour in Sport (n = 1239).

	Prosocial behavior in sports		Antisocial behavior in sports	
	*β*	*t*	*β*	*t*
gender	−0.03	−1.21	−0.10	−3.61^***^
age	0.04	1.82	−0.01	−0.38
Years of physical activity	−0.02	−0.70	−0.01	−0.44
Sports level	0.01	0.27	0.05	1.87
Democratic leadership behavior	0.52	21.60^***^	−0.32	−11.73^***^
F	94.88^***^		29.94^***^	
R^2^	0.28		0.11	
Adj R^2^	0.28		0.11	

**Table 3:** Regression Analysis of Autocratic Leadership Behaviour on Prosocial and Antisocial Behaviour in Sport (n = 1239).

	Prosocial behavior in sports		Antisocial behavior in sports	
	*β*	*t*	*β*	*t*
gender	−0.05	−1.92	−0.09	−3.50^***^
age	0.04	1.51	−0.02	−0.72
Years of physical activity	−0.02	−0.74	−0.02	−0.66
Sports level	0.03	1.01	0.01	0.27
Democratic leadership behavior	−0.30	−10.80^***^	0.51	20.75^***^
F	24.61^***^		89.10^***^	
R^2^	0.09		0.27	
Adj R^2^	0.09		0.26	

### 4.5 The mediating effect test of goal orientation and moral disengagement in coach leadership behavior and sports prosocial behavior

In their 2012 study on mediation effect analysis, Fang et al. [35] found that the bias-corrected percentile Bootstrap method outperforms the Sobel test. This method involves resampling from the original sample while ensuring that each observation unit has an equal probability of being selected in every draw. Accordingly, the mediation analysis was conducted using the Process4.1 plugin, a SPSS 26.0 macro program developed by Hayes [36], while controlling for demographic variables such as gender, age, years of sports participation, and athletic level. A 95% confidence interval for the mediation effect that includes zero indicates a non-significant mediation effect; otherwise, the effect is considered statistically significant.

The regression analysis results (Table 4) show that democratic leadership behavior can significantly negatively predict self-orientation (β = −0.21, P < 0.001); When both democratic leadership behavior and self-orientation predict moral disengagement in sports, democratic leadership behavior has a significant negative predictive effect (β = −0.13, P < 0.001), while self-orientation has a significant positive predictive effect (β = 0.31, P < 0.001); When democratic leadership behavior, self-orientation, and moral disengagement in sports simultaneously predict prosocial behavior in sports, democratic leadership behavior has a significant positive predictive effect (β = 0.48, P < 0.001), while self-orientation and moral disengagement in sports have a significant negative predictive effect (β = −0.11, P < 0.001; β = −0.08, P < 0.01); When democratic leadership behavior, self-orientation, and moral disengagement in sports simultaneously predict antisocial behavior in sports, democratic leadership behavior has a significant negative predictive effect (β = −0.22, P < 0.001), while self-orientation and moral disengagement in sports have a significant positive predictive effect (β = 0.11, P < 0.001, β = 0.36, P < 0.001).

**Table 4:** Regression Analyses of Tests of the Mediating Effects of Ego Orientation and Sport Moral Disengagement on the Relationship Between Democratic Leadership Behaviour and Prosocial and Antisocial Behaviour in Sport (n = 1239).

regression equation		Overall fitting index			Significance of the regression coefficient	
outcome	predictor variable	*R*	*R* ^*2*^	*F*	*β*	*t*
Self-orientation	Democratic leadership behavior	0.22	0.05	12.29	−0.21	−7.70^***^
Moral disengagement in sports	Self-orientation	0.38	0.14	33.74	0.31	11.29^***^
	Democratic leadership behavior				−0.13	−4.93^***^
Prosocial behavior in sports	Self-orientation	0.55	0.30	75.80	−0.11	−4.12^***^
	Moral disengagement in sports				−0.08	−3.24^**^
	Democratic leadership behavior				0.48	19.65^***^
Antisocial behavior in sports	Self-orientation	0.52	0.27	63.68	0.11	4.35^***^
	Moral disengagement in sports				0.36	13.47^***^
	Democratic leadership behavior				−0.22	−8.74^***^

The regression analysis results (Table 5) show that democratic leadership behavior can significantly positively predict task orientation (β = 0.53, P < 0.001); When both democratic leadership behavior and task orientation predict moral disengagement in sports, democratic leadership behavior has a significant negative predictive effect (β = −0.18, P < 0.001), while the predictive effect of task orientation is not significant (β = −0.04, P > 0.05); When democratic leadership behavior, task orientation, and moral disengagement in sports predict prosocial behavior simultaneously, democratic leadership behavior and task orientation have a significant positive predictive effect (β = 0.28, P < 0.001; β = 0.42, P < 0.001), while moral disengagement in sports has a significant negative predictive effect (β = −0.10, P < 0.001); When democratic leadership behavior, task orientation, and moral disengagement in sports simultaneously predict antisocial behavior in sports, democratic leadership behavior and task orientation have a significant negative predictive effect (β = −0.18, P < 0.001; β = −0.11, P < 0.001), while moral disengagement in sports has a significant positive predictive effect (β = 0.39, P < 0.001).

**Table 5:** Regression Analyses of Tests of the Mediating Effects of Task Orientation and Sport Moral Disengagement on the Relationship Between Democratic Leadership Behaviour and Prosocial and Antisocial Behaviour in Sport(n = 1239).

regression equation		Overall fitting index			Significance of the regression coefficient	
outcome	predictor variable	*R*	*R* ^*2*^	*F*	*β*	*t*
task orientation	Democratic leadership behavior	0.53	0.28	94.03	0.53	21.66^***^
Moral disengagement in sports	task orientation	0.23	0.05	11.58	−0.04	−1.17
	Democratic leadership behavior				−0.18	−5.48^***^
Prosocial behavior in sports	task orientation	0.65	0.42	125.55	0.42	16.24^***^
	Moral disengagement in sports				−0.10	−4.63^***^
	Democratic leadership behavior				0.28	10.98^***^
Antisocial behavior in sports	task orientation	0.51	0.26	62.93	−0.11	−3.87^***^
	Moral disengagement in sports				0.39	15.39^***^
	Democratic leadership behavior				−0.18	−6.20^***^

The regression analysis results (Table 6) show that authoritarian leadership behavior can significantly positively predict self-orientation (β = 0.27, P < 0.001); When both authoritarian leadership behavior and self-orientation predict moral disengagement in sports, authoritarian leadership behavior has a significant positive predictive effect (β = 0.31, P < 0.001), and self-orientation also has a significant positive predictive effect (β = 0.25, P < 0.001); When authoritarian leadership behavior, self-orientation, and moral disengagement in sports simultaneously predict prosocial behavior in sports, authoritarianism has a significant negative predictive effect (β = −0.23, P < 0.001), while self-orientation and moral disengagement in sports also have a significant negative predictive effect (β = −0.15, P < 0.001; β = −0.08, P < 0.01); When authoritarian leadership behavior, self-orientation, and moral disengagement in sports simultaneously predict antisocial behavior in sports, authoritarian leadership behavior has a significant positive predictive effect (β = 0.38, P < 0.001), and self-orientation and moral disengagement in sports also have a significant positive predictive effect (β = 0.09, P < 0.001, β = 0.26, P < 0.001).

**Table 6:** Regression Analyses of Tests of the Mediating Effects of Ego Orientation and Sport Moral Disengagement on the Relationship Between Authoritarian Leadership Behaviour and Prosocial and Antisocial Behaviour in Sport(n = 1239).

regression equation		Overall fitting index			Significance of the regression coefficient	
outcome	predictor variable	*R*	*R* ^*2*^	*F*	*β*	*t*
self-orientation	Authoritarian leadership behavior	0.27	0.07	19.46	0.27	9.75^***^
Moral disengagement in sports	Self-orientation	0.46	0.21	55.30	0.25	9.57^***^
	Authoritarian leadership behavior				0.31	11.73^***^
Prosocial behavior in sports	Self-orientation	0.35	0.12	25.01	−0.15	−5.23^***^
	Moral disengagement in sports				−0.08	−2.66^**^
	Authoritarian leadership behavior				−0.23	−7.72^***^
prosocial behavior in sports	Self-orientation	0.59	0.34	91.61	0.09	3.62^***^
	Moral disengagement in sports				0.26	10.07^***^
	Authoritarian leadership behavior				0.38	15.13^***^

The regression analysis results (Table 7) show that authoritarian leadership behavior can significantly negatively predict task orientation (β = −0.22, P < 0.001); When both authoritarian leadership behavior and task orientation predict moral disengagement in sports, authoritarian leadership behavior has a significant positive predictive effect (β = 0.37, P < 0.001), while the predictive effect of task orientation is not significant (β = −0.05, P > 0.05); When authoritarian leadership behavior, task orientation, and moral disengagement in sports simultaneously predict prosocial behavior in sports, authoritarian leadership behavior and moral disengagement in sports have a significant negative predictive effect (β = −0.14, P < 0.001; β = −0.09, P < 0.001), while task orientation has a significant positive predictive effect (β = 0.53, P < 0.001); When authoritarian leadership behavior, task orientation, and moral disengagement in sports simultaneously predict antisocial behavior in sports, authoritarian leadership behavior and moral disengagement in sports have a significant positive predictive effect (β = 0.37, P < 0.001; β = 0.28, P < 0.001), while task orientation has a significant negative predictive effect (β = −0.14, P < 0.001).

**Table 7:** Regression Analyses of Tests of the Mediating Effects of Task Orientation and Sport Moral Disengagement on the Relationship Between Authoritarian Leadership Behaviour and Prosocial and Antisocial Behaviour in Sport(n = 1239).

regression equation		Overall fitting index			Significance of the regression coefficient	
outcome	predictor variable	*R*	*R* ^*2*^	*F*	*β*	*t*
task orientation	Authoritarian leadership behavior	0.23	0.05	13.21	−0.22	−8.06^***^
Moral disengagement in sports	Self-orientation	0.40	0.16	37.95	−0.05	−1.88
	Authoritarian leadership behavior				0.37	13.55^***^
Prosocial behavior in sports	Self-orientation	0.61	0.38	105.96	0.53	23.12^***^
	Moral disengagement in sports				−0.09	−3.72^***^
	Authoritarian leadership behavior				−0.14	−5.72^***^
antisocial behavior in sports	Self-orientation	0.59	0.35	96.11	−0.14	−5.84^***^
	Moral disengagement in sports				0.28	11.20^***^
	Authoritarian leadership behavior				0.37	14.66^***^

The intermediary effect test Bootstrap The analysis results (Table 8) show that:

**Table 8:** Bootstrap Analyses for the Mediated Effects Test (n = 1239).

assume	route	Indirect effect value	LLCI	ULCI	Direct effect value	LLCI	ULCI	Test results
H2a	Democratic leadership behavior → self-orientation → prosocial behavior in sports	0.021	0.009	0.035	0.434	0.391	0.478	establish
H3a	Democratic leadership behavior → moral disengagement in sports → prosocial behavior in sports	0.010	0.003	0.020				establish
H4a	Democratic leadership behavior → self-orientation → moral disengagement in sports → prosocial behavior in sports	0.005	0.001	0.010				establish
H2b	Democratic leadership behavior → task orientation → prosocial behavior in sports	0.197	0.161	0.238	0.254	0.209	0.300	establish
H3a	Democratic leadership behavior → moral disengagement in sports → prosocial behavior in sports	0.017	0.007	0.029				establish
H4b	Democratic leadership behavior → task orientation → moral disengagement in sports → prosocial behavior in sports	0.002	−0.002	0.006				Not establish
H2c	Democratic leadership behavior → self-orientation → Movement antisocial behavior	−0.017	−0.029	−0.008	−0.155	−0.190	−0.121	establish
H3b	Democratic leadership behavior → moral disengagement in sports → antisocial behavior in sports	−0.033	−0.052	−0.017				establish
H4c	Democratic leadership behavior → self-orientation → moral disengagement in sports → antisocial behavior in sports	−0.016	−0.024	−0.010				establish
H2d	Democratic leadership behavior → Task orientation → Movement antisocial behavior	−0.041	−0.069	−0.014	−0.127	−0.167	−0.087	establish
H3b	Democratic leadership behavior → moral disengagement in sports → antisocial behavior in sports	−0.049	−0.074	−0.027				establish
H4d	Democratic leadership behavior → task orientation → moral disengagement in sports → antisocial behavior in sports	−0.005	−0.016	0.006				Not establish
H2e	Authoritarian leadership behavior → self-orientation → prosocial behavior in sports	−0.041	−0.061	−0.023	−0.237	−0.296	−0.179	establish
H3c	Authoritarian leadership behavior → moral disengagement in sports → prosocial behavior in sports	−0.025	−0.047	−0.004				establish
H4e	Authoritarian leadership behavior → self-orientation → moral disengagement in sports → prosocial behavior in sports	−0.005	−0.011	−0.001				establish
H2f	Authoritarian leadership behavior → task orientation → prosocial behavior in sports	−0.122	−0.166	−0.082	−0.151	−0.201	−0.102	establish
H3c	Authoritarian leadership behavior → moral disengagement in sports → prosocial behavior in sports	−0.034	−0.058	−0.012				establish
H4f	Authoritarian leadership behavior → task orientation → moral disengagement in sports → prosocial behavior in sports	−0.001	−0.003	0.001				Not establish
H2g	Authoritarian leadership behavior → self-orientation → Movement antisocial behavior	0.020	0.008	0.034	0.299	0.260	0.339	establish
H3d	Authoritarian leadership behavior → moral disengagement in sports → antisocial behavior in sports	0.066	0.043	0.090				establish
H4g	Authoritarian leadership behavior → self-orientation → moral disengagement in sports → antisocial behavior in sports	0.014	0.009	0.021				establish
H2h	Authoritarian leadership behavior → Task orientation → Movement antisocial behavior	0.025	0.013	0.041	0.289	0.249	0.328	establish
H3d	Authoritarian leadership behavior → moral disengagement in sports → antisocial behavior in sports	0.083	0.057	0.112				establish
H4h	Authoritarian leadership behavior → task orientation → moral disengagement in sports → antisocial behavior in sports	0.002	−0.001	0.006				Not establish

(1) After introducing the mediating variables of self-orientedness and sports moral disengagement between democratic leadership behavior and sports prosocial behavior, the mediating effect value of self-orientedness was 0.021, with a confidence interval of [0.009, 0.035] and not including 0. The mediating effect value of sports moral disengagement was 0.010, with a confidence interval of [0.003, 0.020] and not including 0. The chain mediating effect value of self-orientedness and sports moral disengagement was 0.005, with a confidence interval of [0.001, 0.010] and not including 0. Democratic leadership behavior has a significant positive impact on sports prosocial behavior, with a direct effect value of 0.434, and a confidence interval of [0.391, 0.478]. This indicates that both self-orientedness and sports moral disengagement have independent partial mediating effects between democratic leadership behavior and sports prosocial behavior, and the chain mediating effect of self-orientedness and sports moral disengagement also exists. Therefore, assuming H2a、H3a、H4a all are established.
(2) After introducing the mediating variables of task orientation and sports moral disengagement between democratic leadership behavior and sports prosocial behavior, the mediating effect value of task orientation was 0.197, with a confidence interval of [0.161, 0.238] and not including 0. The mediating effect value of sports moral disengagement was 0.017, with a confidence interval of [0.007, 0.029] and not including 0. The chained mediating effect value of task orientation and sports moral disengagement was 0.002, with a confidence interval of [−0.002, 0.006] including 0. Democratic leadership behavior has a significant positive impact on sports prosocial behavior, with a direct effect value of 0.254, and a confidence interval of [0.209, 0.300]. This further verifies that sports moral disengagement has a partial mediating effect between democratic leadership behavior and sports prosocial behavior. At the same time, it indicates that task orientation has a partial mediating effect between democratic leadership behavior and sports prosocial behavior, while the chained mediating effect of task orientation and sports moral disengagement does not exist.
(3) After introducing the mediating variables of self-orientedness and sports moral evasion between democratic leadership behavior and sports antisocial behavior, the mediating effect value of self-orientedness was −0.017, with a confidence interval of [−0.029, −0.008] and not including 0. The mediating effect value of sports moral evasion was −0.033, with a confidence interval of [−0.052, −0.017] and not including 0. The chain mediating effect value of self-orientedness and sports moral evasion was −0.016, with a confidence interval of [−0.024, −0.010] and not including 0. Democratic leadership behavior has a significant negative impact on sports antisocial behavior, with a direct effect value of −0.155, and a confidence interval of [−0.190, −0.121]. This indicates that both self-orientedness and sports moral evasion have independent partial mediating effects between democratic leadership behavior and sports antisocial behavior, and the chain mediating effect of self-orientedness and sports moral evasion also exists. Therefore, assuming H2c、H3b、H4c are all established.
(4) After introducing the mediating variables of task orientation and sports moral evasion between democratic leadership behavior and sports antisocial behavior, the mediating effect value of task orientation was −0.041, with a confidence interval of [−0.069, −0.014] and not including 0. The mediating effect value of sports moral evasion was −0.049, with a confidence interval of [−0.074, −0.027] and not including 0. The chained mediating effect value of task orientation and sports moral evasion was −0.005, with a confidence interval of [−0.016, 0.006] which includes 0. Democratic leadership behavior has a significant negative impact on sports antisocial behavior, with a direct effect value of −0.127, and a confidence interval of [−0.167, −0.087]. This further verifies that sports moral evasion has a partial mediating effect between democratic leadership behavior and sports antisocial behavior. At the same time, it indicates that task orientation has a partial mediating effect between democratic leadership behavior and sports antisocial behavior, while the chained mediating effect of task orientation and sports moral evasion does not exist. Therefore, assuming H2d is established, assuming H4d is not established.
(5) After introducing the mediating variables of self-orientedness and sports moral disengagement between autocratic leadership behavior and sports prosocial behavior, the mediating effect value of self-orientedness was −0.041, with a confidence interval of [−0.061, −0.023] and not including 0. The mediating effect value of sports moral disengagement was −0.025, with a confidence interval of [−0.047, −0.004] and not including 0. The chain mediating effect value of self-orientedness and sports moral disengagement was −0.005, with a confidence interval of [−0.011, −0.001] and not including 0. Autocratic leadership behavior has a significant negative impact on sports prosocial behavior, with a direct effect value of −0.237, and a confidence interval of [−0.296, −0.179]. This indicates that both self-orientedness and sports moral disengagement have independent partial mediating effects between autocratic leadership behavior and sports prosocial behavior, and the chain mediating effect of self-orientedness and sports moral disengagement also exists. Therefore, assuming H2e、H3c、H4e are all established.
(6) After introducing the mediating variables of task orientation and sports moral disengagement between autocratic leadership behavior and sports prosocial behavior, the mediating effect value of task orientation was −0.122, with a confidence interval of [−0.166, −0.082] and not including 0. The mediating effect value of sports moral disengagement was −0.034, with a confidence interval of [−0.058, −0.012] and not including 0. The chained mediating effect value of task orientation and sports moral disengagement was −0.001, with a confidence interval of [−0.003, 0.001] which includes 0. Autocratic leadership behavior has a significant negative impact on sports prosocial behavior, with a direct effect value of −0.151, and a confidence interval of [−0.201, −0.102]. This further verifies that sports moral disengagement has a partial mediating effect between autocratic leadership behavior and sports prosocial behavior. At the same time, it indicates that task orientation has a partial mediating effect between autocratic leadership behavior and sports prosocial behavior, while the chained mediating effect of task orientation and sports moral disengagement does not exist.Therefore, assuming H2f is established, assuming H4f is not established.
(7) After introducing the mediating variables of self-orientedness and sports moral evasion between autocratic leadership behavior and sports antisocial behavior, the mediating effect value of self-orientedness was 0.020, with a confidence interval of [0.008, 0.034] and not including 0. The mediating effect value of sports moral evasion was 0.066, with a confidence interval of [0.043, 0.090] and not including 0. The chain mediating effect value of self-orientedness and sports moral evasion was 0.014, with a confidence interval of [0.009, 0.021] and not including 0. Autocratic leadership behavior has a significant positive impact on sports antisocial behavior, with a direct effect value of 0.299, and a confidence interval of [0.260, 0.339]. This indicates that both self-orientedness and sports moral evasion have independent partial mediating effects between autocratic leadership behavior and sports antisocial behavior, and the chain mediating effect of self-orientedness and sports moral evasion also exists. Therefore, assuming H2g、H3d、H4g are all established.
(8) After introducing the mediating variables of task orientation and sports moral disengagement between autocratic leadership behavior and sports antisocial behavior, the mediating effect value of task orientation was 0.025, with a confidence interval of [0.013, 0.041] and not including 0. The mediating effect value of sports moral disengagement was 0.083, with a confidence interval of [0.057, 0.112] and not including 0. The chained mediating effect value of task orientation and sports moral disengagement was 0.002, with a confidence interval of [−0.001, 0.006] including 0. Autocratic leadership behavior has a significant positive impact on sports antisocial behavior, with a direct effect value of 0.289, and a confidence interval of [0.249, 0.328]. This further verifies that sports moral disengagement has a partial mediating effect between autocratic leadership behavior and sports antisocial behavior. At the same time, it indicates that task orientation has a partial mediating effect between autocratic leadership behavior and sports antisocial behavior, while the chained mediating effect of task orientation and sports moral disengagement does not exist. Therefore, assuming H2h is established, assuming H4h is not established.

## 5 Discussions

### 5.1 The direct effect of coach leadership behavior on pro-antisocial behavior in sports

Research has found that coach democratic leadership behavior can positively predict prosocial behavior in sports and negatively predict antisocial behavior in sports; Coach authoritarian leadership behavior can positively predict antisocial behavior in sports and negatively predict prosocial behavior in sports. According to social cognitive theory, coaches play a significant role in the social behavior of athletes during training and competition [16]. Democratic coaches will take into account the ideas of the athletes when formulating training plans and competition strategies, and will show respect and encouragement to the athletes. When coaches and athletes participate in the decision-making process together, it enhances communication among team members, promotes emotional exchanges among athletes, reduces the occurrence of conflicts or negative behaviors among team members, improves the quality of relationships among athletes, and at the same time, the positive factors such as the coaches’ respect and encouragement for the athletes help to set a good example for the athletes, which is conducive to the formation of the athletes’ sense of responsibility and the shaping of their social responsibility. It is also more conducive to the cultivation of their professional skills and the improvement of their sports performance. As a result, athletes are more likely to exhibit pro-social behaviors during training and competitions and reduce anti-social behaviors. Just like the coach Phil Jackson, who led the team to win 11 NBA championships, he gave the players full respect and the right to speak, which made the relationship between the coach and the players very harmonious. At the same time, players understood and respected each other, which enabled them to achieve miracles on the court [37]. Related studies have found that democratic behavior of coaches and parents can effectively promote the formation of individual social responsibility, while authoritarian behavior can effectively inhibit the formation of individual social responsibility [16,17]. Autocratic coaches are self-centered, make strong decisions, and do not allow athletes to participate in competition training decisions. They often treat athletes with commands, criticism, punishment, blame, and indifference. This goes against the training relationship advocated by competitive sports, which is “coach-led, athlete-led, and win-win cooperation.” It will lead to a lack of trust and communication between coaches and athletes, as well as a lack of emotional interaction between athletes. This can easily result in opposing emotions and conflicts within the team. At the same time, it will increase athletes’ psychological pressure, lack confidence, and fear of criticism and failure, leading to negative emotions during intense training and competition. In order to release the oppression and grievances in their hearts, and at the same time, in order to win the competition, athletes often neglect their social responsibility and engage in more antisocial behaviors during sports.

Although different coach leadership behaviors can significantly predict athletes’ prosocial and antisocial behaviors in sports, different coach leadership behaviors have different explanatory powers for athletes’ prosocial and antisocial behaviors in sports. The explanatory power of democratic leadership behavior for prosocial behavior and antisocial behavior in sports is 28% and 11%, respectively; The explanatory power of authoritarian leadership behavior for prosocial behavior and antisocial behavior in sports is 9% and 26%, respectively. Through comparative analysis, it was found that democratic leadership behavior has a higher explanatory power for prosocial behavior in sports than for antisocial behavior in sports, while authoritarian leadership behavior has a higher explanatory power for antisocial behavior in sports than for prosocial behavior in sports. The reason for this result might be that democratic leadership behavior affirmed the dominant position of athletes in training, created an atmosphere of equal interaction in training management, gave athletes full respect and the right to speak, allowing athletes to feel the warmth of “home” within the team, and providing them with more positive emotional value. The nature of sports prosocial behavior is an active behavior that conforms to moral norms, while the nature of sports antisocial behavior is a passive behavior that violates moral norms. Authoritarian leadership behavior essentially represents the behavior of a coach controlling athletes, depriving athletes of the autonomy in training and competitions, and failing to pay attention to and meet their psychological needs. This is likely to lead to mutual accusations, suspicions, and complaints between the coach and the athlete, and subsequently, the accumulation of negative emotions for the athlete. Therefore, democratic leadership behavior has a higher explanatory power for sports prosocial behavior, while authoritarian leadership behavior has a higher explanatory power for sports antisocial behavior.

### 5.2 The mediating role of goal orientation between coach leadership behavior and sports prosocial behavior

Research has found that goal orientation partially mediates the relationship between coach leadership behavior and athlete sports prosocial behavior. Coach democratic leadership behavior can increase athletes’ task orientation, reduce their self-orientation, thereby increasing their prosocial behavior in sports and reducing their antisocial behavior in sports; Coach authoritarian leadership behavior can reduce athletes’ task orientation, increase their self-orientation, thereby reducing their prosocial behavior in sports and increasing their antisocial behavior in sports. Research has shown that coach behavior is an important factor influencing athletes’ goal orientation [38] goal orientation can directly affect athletes’ pro-antisocial behavior in sports [1]. On the one hand, democratic leadership behavior of coaches can promote athletes’ task goal orientation and inhibit their self-orientation; On the other hand, authoritarian leadership of coaches behavior can inhibit athletes’ task goal orientation and promote self-orientation. From the perspective of goal theory, the situation created by coaches is called motivational atmosphere, which can affect the probability of athletes’ self-participation or task participation in sports activities [39]. Democratic coaches allow and encourage athletes to participate in training and competition decision-making, emphasize task mastery and competition quality, and have less aggressive language towards athletes [22,23]. This reflects the concept of athlete development as the center, promotes communication and exchange between coaches and athletes, improves the quality of the relationship between coaches and athletes, and is conducive to guiding athletes to show stronger collectivism, thereby creating a task-oriented atmosphere for athletes, reducing the formation of individualism and cognitive bias towards self-improvement. Syrmpas and Bekiari [22] found that democratic coaches tend to have task-oriented leadership. However, coaches with authoritarian leadership behavior do not allow athletes to participate in decision-making and require them to execute in a commanding manner, while also using more aggressive language towards athletes [40]. Athletes do not feel respected and valued by coaches, which increases their sense of frustration and psychological pressure. This leads athletes to believe that success is about defeating opponents during training and competition, establishing advantages that are beneficial to their own training and competition, and ultimately achieving victory in the competition, rather than cooperating with others to improve their own skills. At the same time, it also makes athletes believe that only success can reduce their sense of failure [22].

On the other hand, the enhancement of task orientation and the weakening of self-orientation will make individuals focus on learning as their goal, and conceive success and judge their own abilities based on their own efforts and personal progress. Specifically for athletes, they tend to focus on mastering and improving their own sports skills, hoping to experience fair competition during training and competitions. This reduces their own sports anxiety and makes them more empathetic towards others, leading to a tendency for athletes to think from others’ perspectives and increase their prosocial behavior while reducing their antisocial behavior [41]. Individuals with weakened task orientation and enhanced self-orientation tend to establish a sense of superiority by comparing themselves with others. Athletes believe that success requires mastering sports skills and achieving better sports performance than others, which enhances their utilitarian mindset in training and competition environments, increases their recognition of intentional injury behavior, leads to a decrease in athletes’ prosocial behavior in sports, and an increase in their antisocial behavior in sports [2].

### 5.3 The mediating role of ethical disengagement in the relationship between coach leadership behavior and pro-antisocial behavior in sports

Ethical disengagement in sports; Coach authoritarian leadership behavior can reduce athletes’ prosocial behavior and increase their antisocial behavior by increasing moral disengagement in sports. Democratic leadership coaches are good at creating a democratic training atmosphere, willing to listen to athletes’ opinions, respect athletes, tailor training goals and career plans for athletes, meet their internal needs, set a moral example for athletes, promote the formation of athletes’ sense of responsibility and the shaping of athletes’ social responsibility, and make them understand that respecting opponents, complying with competition rules, and fair competition are more important than competition results [42]. At the same time, when coaches and athletes discuss decisions, it is beneficial to promote emotional interaction among team members and enhance athletes’ team awareness of unity and cooperation. However, coaches with authoritarian leadership emphasize command and execution in training and competition, and rely on their own decision-making to achieve training goals, with less consideration for athletes’ feelings and psychological tolerance, which limits their subjective initiative and fails to meet their psychological needs. This can easily lead to athletes using training and competition to release their negative emotions, thereby ignoring the norms of sports moral behavior and increasing their level of moral disengagement in sports.

This study found that the relationship between moral disengagement in sports and athletes’ prosocial behavior in sports is significantly positively predicted by moral disengagement in sports, while it significantly negatively predicts prosocial behavior in sports. The research results are consistent with previous studies [10,30]. However, some studies suggest that the relationship between ethical disengagement in sports and prosocial behavior in sports cannot significantly predict prosocial behavior in sports [25,26]. The reason for the differences lies in the fact that athletes with high levels of moral disengagement in sports often use methods such as responsibility transfer, attribution, distortion of consequences, and dispersion of responsibility [25], which can make athletes believe that harmful behavior can be used as a means to help their own team or shift responsibility to coaches. This can also lower the level of moral identity of athletes, leading to less consideration for respecting opponents in training and competition, and thus less prosocial behavior in sports, and more antisocial behavior in sports. On the other hand, athletes with low levels of moral disengagement in sports believe that antisocial behavior in sports goes against the sportsmanship of unity, respect for opponents, and fairness. At the same time, athletes with higher levels of moral identification are more likely to engage in prosocial behavior in sports and less likely to engage in antisocial behavior.

### 5.4 The chain mediating effect of goal orientation and sports moral deduction between coach leadership behavior and sports prosocial behavior

Both coach democratic leadership behavior and authoritarian leadership behavior can influence athletes’ prosocial and antisocial behavior in sports through a chain mediation of self-orientation and moral disengagement; Both coach democratic leadership behavior and authoritarian leadership behavior can significantly predict athletes’ sports prosocial behavior and sports antisocial behavior through the independent mediating effect of task orientation and sports ethics deduction, but neither can affect athletes’ sports prosocial behavior and sports antisocial behavior through the chain mediating effect of task orientation and sports ethics deduction. Coach’s democratic leadership behavior can make athletes realize that their coach is an intimate and emotional coach, which can enhance their desire to learn and communicate. Athletes are willing to make greater efforts to improve their athletic abilities and knowledge reserves, which is conducive to creating a task-oriented atmosphere, but not conducive to forming a self-oriented atmosphere. This makes athletes truly understand that achieving success is not about striving to defeat others and have superior abilities, but about learning and collaborating with others [43]. On the one hand, when the task orientation is the main focus, athletes tend to respect the sportsmanship of unity, cooperation, respect for opponents, and fairness, and tend to focus on mastering and improving their own skills. They hope to experience fair competition in training and competition, and are willing to help others in the sports environment, deliberately avoiding moral disengagement in sports [10]. On the other hand, when self-orientation decreases, it means that athletes’ utilitarianism and sports anxiety will decrease, making it easier for them to remain calm and composed during training and competition, which is beneficial for reducing their negative emotions. At the same time, there will be more attention paid to the requirements of sports fairness and justice, thereby reducing their level of moral disengagement in sports. The reduction of athletes’ level of moral disengagement in sports will improve their level of moral identity. During training and competition, athletes will put themselves in others’ shoes, consider respecting their opponents more, and help and cooperate with their teammates. At the same time, they will also use less moral disengagement in sports to justify their moral misconduct during competition, thereby increasing prosocial behavior in sports and reducing antisocial behavior in sports [8].

Coach’s authoritarian leadership behavior makes athletes perceive themselves as a coach who is accustomed to training methods such as punishment, negative communication, and commands. This can easily create an atmosphere of distrust and insecurity within the team, leading to increased psychological pressure, fatigue, lack of confidence, and fear of failure among athletes. This can result in a deviation in personal understanding and cognition of success and competition, leading athletes to believe that success is about surpassing others, and competition is about using any means necessary to achieve victory, rather than improving their own abilities and cooperating with others [43]. In other words, it can easily lead to an increase in athletes’ self-orientation and a decrease in their task orientation. When self-orientation is dominant, there is no deliberate avoidance of moral disengagement in sports, which means that athletes’ internal motivation will also be enhanced, which can easily lead to athletes being willing to use cheating and deception to achieve their personal goals, increasing in athletes’ level of moral disengagement in sports [44,45]. The increase in athletes’ level of moral disengagement in sports will lead to a decrease in their level of moral identity, less empathy in training and competition, and less consideration for respecting opponents. At the same time, they will also use more sports moral disengagement mechanisms to justify their moral misconduct during competition, thereby reducing sports prosocial behavior and increasing sports antisocial behavior [8].

Therefore, democratic leadership behavior of the coach can reduce the level of athletes’ evasion of sports morality, making them less likely to engage in anti-social sports behaviors and more likely to exhibit prosocial sports behaviors; autocratic leadership behavior can enhance the self-oriented behavior of athletes, thereby increasing the level of evasion of sports morality among them, making them less likely to exhibit prosocial sports behaviors and more likely to exhibit anti-social sports behaviors; democratic leadership behavior can only achieve the effect of reducing the level of evasion of sports morality among athletes by promoting their task-oriented behavior, but not by promoting task-oriented behavior to further reduce the level of evasion of sports morality, thereby increasing prosocial sports behaviors and reducing anti-social sports behaviors of athletes; autocratic leadership behavior can only achieve the effect of increasing the level of evasion of sports morality among athletes by suppressing their task-oriented behavior, but not by suppressing task-oriented behavior to further increase the level of evasion of sports morality, thereby reducing prosocial sports behaviors and increasing anti-social sports behaviors of athletes.

### 5.5 Research significance and limitations

This study explores the relationship between coach leadership behavior and athlete sports prosocial behavior, expands the research attributes of coach leadership behavior and athlete sports prosocial behavior, enriches the formation mechanism of goal orientation and sports moral disengagement, and discovers the internal mechanism of goal orientation and sports moral disengagement between coach leadership behavior and athlete sports prosocial behavior. It helps coaches engaged in physical education teaching and training to understand the possible impact of leadership behavior on athletes sports prosocial behavior, and better apply relevant knowledge to improve athletes moral behavior norms and solve the problem of athlete sports moral behavior misconduct. However, this article also has certain research limitations: (1) Collecting data through scales can easily lead to subjective influence on the validity of the data by the participants. (2) Due to the use of a cross-sectional study, it is impossible to infer the causal relationship between the variables. (3) Only considering the mediating effect of goal orientation and ethical disengagement in the relationship between coach leadership behavior and athlete pro-antisocial behavior in sports, but it cannot be ruled out that there are other mediating variables, such as coach-athlete relationships, effective communication within the team, etc., all of which require further in-depth research.

## 6 Conclusion

(1) Coach democratic leadership behavior has a significant positive impact on athletes’ prosocial behavior in sports and a significant negative impact on their antisocial behavior in sports; Coach authoritarian leadership behavior has a significant negative impact on athletes’ prosocial behavior in sports and a significant positive impact on their antisocial behavior in sports. This indicates that coach democratic leadership behavior has a suppressive effect on athletes’ antisocial behavior in sports and a promoting effect on sports’ prosocial behavior; Authoritarian leadership behavior has a promoting effect on athletes’ antisocial behavior in sports and a restraining effect on their prosocial behavior in sports.
(2) Both coach democratic leadership behavior and authoritarian leadership behavior can directly affect athletes’ prosocial behavior and antisocial behavior in sports, and can also indirectly affect athletes’ prosocial behavior and antisocial behavior in sports through the mediating effects of task orientation, self-orientation, and sports ethics deduction. Specific mediating paths include independent mediating paths of self-orientation, task orientation, and sports ethics deduction, and chain mediating paths of self-orientation and sports ethics deduction. This indicates that coach democratic leadership behavior can lower athletes’ level of moral disengagement by suppressing their self-orientation, making them less likely to engage in antisocial behavior and more likely to engage in prosocial behavior; Authoritarian leadership behavior can enhance athletes’ level of moral disengagement in sports by promoting self-orientation, making them less likely to engage in prosocial sports behavior and more likely to engage in antisocial sports behavior; Democratic leadership behavior can only reduce the level of moral disengagement in sports by promoting task orientation among athletes, but cannot reduce the level of moral disengagement in sports by promoting task orientation, causing athletes to increase their prosocial behavior in sports and reduce their antisocial behavior in sports; Authoritarian leadership behavior can only improve athletes’ moral disengagement by inhibiting their task orientation, but cannot improve athletes’ moral disengagement by inhibiting their task orientation, reducing their prosocial behavior and increasing their anti social behavior.
